# Atrazine Exposure Induces Hepatic Metabolism Disorder in Male Adult Zebrafish

**DOI:** 10.3390/toxics10070400

**Published:** 2022-07-19

**Authors:** Hu Zhang, Xiaofang Wang, Mingrong Qian, Yuanxiang Jin

**Affiliations:** 1 Zhejiang Province Key Laboratory for Food Safety, Institute of Agro-Product Safety and Nutrition, Zhejiang Academy of Agricultural Sciences, Hangzhou 310021, China; zhanghu@mail.zaas.ac.cn; 2 Key Laboratory of Pollution Exposure and Health Intervention of Zhejiang Province, Interdisciplinary Research Academy, Zhejiang Shuren University, Hangzhou 310015, China; wangxiaofang0811@163.com; 3 College of Biotechnology and Bioengineering, Zhejiang University of Technology, Hangzhou 310032, China

**Keywords:** atrazine, hepatic metabolism, zebrafish, metabolomic

## Abstract

Atrazine (ATZ) is a herbicide used in agricultural production and has been detected in surface water due to its widespread use worldwide. This may pose a threat to the health of aquatic animals. To explore the ATZ−induced hepatic metabolism disorder, male zebrafish were exposed to 300 and 1000 μg/L ATZ for 21 days, respectively. The results revealed that ATZ exposure significantly reduced hepatic triglyceride (TG) levels, while significantly (*p* < 0.05) increased pyruvate (PYR) and total cholesterol (TC) levels. In addition, the liver sample from the 1000 μg/L ATZ−treated group was used for GC/MS metabolomic analysis. The principal component analysis (PCA) model showed significant separation of the 1000 μg/L ATZ group from the control group, indicating that ATZ exposure altered hepatic metabolism in male adult zebrafish. A total of 29 significantly (*p* < 0.05) different metabolites were observed and identified in the ATZ−treated group. Moreover, the most disturbed pathways by ATZ were the arginine and proline metabolic pathways, followed by the glutathione metabolic pathway. Three and two metabolites were significantly altered in the arginine and proline metabolic pathways and glutathione metabolic pathway, respectively. Based on these results, we suggested that ATZ was capable of altering liver metabolism in zebrafish and that its ecological risk to aquatic organisms cannot be ignored.

## 1. Introduction

In the last decades, pesticide have been widely used to improve the production in agriculture all over the world. Widely used pesticides are present in our daily life [1]. The harm of low concentration pesticide residues to humans and animals can be ignored, but with the improvement of analytical methods, more and more experiments prove that pesticide residues in the environment exceed the standard [2,3]. Pesticide residue hazards have become a growing concern. These residual pesticides can infiltrate into surface water and pose a serious threat to aquatic life [4]. In recent years, many studies have proven that pesticides can affect the water environment [5,6,7].

Atrazine (2−chloro−4−ethylamino−6−isopropylamino−s−triazine, ATZ) has been extensively used as an effective herbicide for the past 50 years. Over the past few decades, 64–80 million pounds of ATZ have been reported to be used annually for agriculture and residential purposes in the United States [8,9]. ATZ in excess of specified standard concentrations has been detected in surface waters in a number of countries [10,11]. Due to the extensive and long-term application of ATZ, the water environment is generally polluted, and the environmental concentration is expected to reach 2.667 mg/L [12]. Growing evidence has recently shown ATZ to have different adverse effects on the endpoint of DNA damage [13], oxidative stress [14,15,16], behavior [17], endocrine disruption [18] and immune toxicity [19] in zebrafish and other aquatic organisms. In animals, glutathione transferases (GSTs) and cytochrome P450, which are responsible for metabolism, are located in the liver and can metabolize ATZ to a variety of products, including desisopropyl atrazine (DIP), desethyl atrazine (DE), atrazine-glutathione (ATZ-SG), atrazine−mercapturate (ATZ−mercap) and diaminochlorotriazine (DACT) [20,21]. On the other hand, the liver is also the primary organ for metabolism. Environmental chemical exposure was shown to cause hepatotoxicity and metabolism disorder in zebrafish [22].

In some studies, metabolomics have been found to be a good explanation for environmental chemical and pesticide toxicity [23,24,25]. Metabolomics systematically studies the metabolism in biological systems, from small cells and tissues to large organs and organisms [26]. GC/MS is a commonly used analytical method for analyzing metabolomics [27]. Because of its high sensitivity and excellent analytical power [28], zebrafish has been widely adopted as an experimental model for environmental toxicity analysis [29,30]. In zebrafish, the liver was considered as the first detoxification organ and some environmental chemicals could be degraded in the liver. Although the toxicity of ATZ has been extensively analyzed in the past decade, the effect of ATZ on hepatic metabolism remains to be explored. Here, male adult zebrafish were exposed to different concentrations of ATZ for 21 days, and at the end of the experiment, it was investigated whether ATZ was able to disrupt hepatic metabolism. We observed that ATZ could disturb several metabolomics pathways in the liver. Additionally, we hope that the results obtained in this study will provide some new information on the toxicity of ATZ to aquatic organisms. 

## 2. Materials and Methods

### 2.1. Chemicals

Atrazine (ATZ, CAS No.: 1912−24−9, purity: >97%) was purchased from TCI and used as received. ATZ was dissolved with dimethyl sulfoxide (DMSO) to prepare stock solutions at concentrations of 300 and 1000 µg/L. The stock solution was kept in reserve at 4 °C until use.

### 2.2. Fish Exposure and Experimental Design

Mature male adult AB strain zebrafish (*Danio rerio*) were used for the present experiment. Experimental zebrafish were acclimatized in tap water (pH 7.0 to 7.5, hardness approximately 50 mg CaCO_3_/L) for at least 1 week, with water temperature controlled at 28 °C and a dark/light photoperiod of 10:14 h [31]. Then, the zebrafish were exposed to ATZ at the concentrations of 300 (ATZ−L) and 1000 μg/L (ATZ−H) with 0.01% DMSO in water for 21 days; 21 days was a relatively common short-term exposure time for zebrafish, and many previous studies also used 21 days as the exposure time [32]. Because of the low toxicity of ATZ to fish, the concentrations used in this study were based on a previous study as a reference [15]. The control group was reared in water with 0.01% DMSO. During the ATZ exposure period, fish were guaranteed to be fed twice daily and water changed every other day. No fish died during the exposure period.

For the hepatic biochemical index analysis, there are three groups: control, ATZ−L (300 μg/L) and ATZ−H (1000 μg/L). Each group of 18 adult males was housed equally in 3 tanks with 3 L of solution in each glass tank. After 21 days exposure, livers from 3 fish were collected together as one sample, resulting in 6 pooled samples per group for further analysis.

To explore the metabolomic analysis of zebrafish liver, experimental exposures were manipulated with reference to our previous studies [33,34]. Briefly, a control group and a treatment group (1000 μg/L) were set up in this part. A total of 84 male adult fish (7 fish per tank, 12 separate glass tanks in total) were exposed to 1000 μg/L ATZ for 21 days. At the same time, the same number of fish were kept in water containing 0.01% DMSO as a control. At the end of the experimental exposure, the livers collected from 14 fish in each group (2 tanks) comprised one sample and 6 pooled samples were collected for analysis in each of the ATZ and control groups. The liver samples were rapidly collected and stored on dry ice and subsequently stored in a −80 °C freezer for further analysis. In the present study, all the fish were anesthetized on ice before dissection.

### 2.3. Determination of Liver Parameters

The liver and PBS were ground at a ratio of 1:9, then centrifuged at 2000 rpm for 10 min and the supernatant was finally collected. Kits for the determination of hepatic glucose (GLU), PYR, TG and TC levels were purchased from Nanjing Jiancheng Biotechnology Research (Nanjing, China) and quantified according to the manufacturer’s designation instructions.

### 2.4. GC/MS-Based Metabolomic Analysis

About 25 mg liver tissue with 1000 µL of acetonitrile/isopropanol/water (3/3/2, *v*/*v*/*v*) with 5 µL 3 mg/mL Myristic acid-d27 and homogenized at low temperature using Tissuelyser-192 (Shanghai Jing Technology Companies, Shanghai, China). The mixture was then placed in an ice bath for 1 min and then centrifuged at 14,000 rpm for 5 min at 4 °C. Then, 800 μL of supernatant was added to the GC vial and evaporated to dryness. A further 20 µL of methylhydroxylamine hydrochloride (40 mg/mL in pyridine) was added and placed in an oscillating incubator at 30 °C for 90 min. Next, 90 µL of MSTFA (N−methyl−N− (trimethylsilyl) trifluoroacetamide) (with 1% TMCS as catalyst) was added and the mixture was incubated at 37 °C for 30 min, followed by centrifugation at 14,000 rpm for 5 min at 4 °C. Finally, 60 µL of supernatant was removed from each sample for GC/MS analysis.

Next, the samples were injected into the Agilent 7890A/Agilent 5975C MSD GC/MS system equipped with an Agilent DB5-MS (30 m × 0.25 mm, 0.25 µm) column. The injection temperature, ion source temperature and transmission line temperature required for the experiment were all set to 250 °C. The helium carrier gas flow rate is set at a constant rate of 1.1 mL/min. Started with a setting of 60 °C and held for 1 min, then increased the speed to 3000 rpm and ran for 10 min. Full scan monitoring was performed over a mass slope range of *m*/*z* 50–500. The GC/MS data were analyzed using ChromaTOF software (v 4.34, LECO, St Joseph, MI, USA). Data analysis was carried out using SIMCA-P+14.0 software (Umetrics, Umea, Sweden).

### 2.5. Data Analysis

All differences between the control and ATZ treatment groups were assessed using a one-way ANOVA. A probability (*p*) value set at 0.05 was deemed to be significant. Graphing was performed using SPSS (Statistical Package for the Social Sciences) 13.0 (SPSS, Chicago, IL, USA) and GraphPad Prism Version 8.0 (GraphPad Software, San Diego, America).

## 3. Results

### 3.1. Effect of ATZ on Physiological Indicators in the Liver

**Figure 1:**
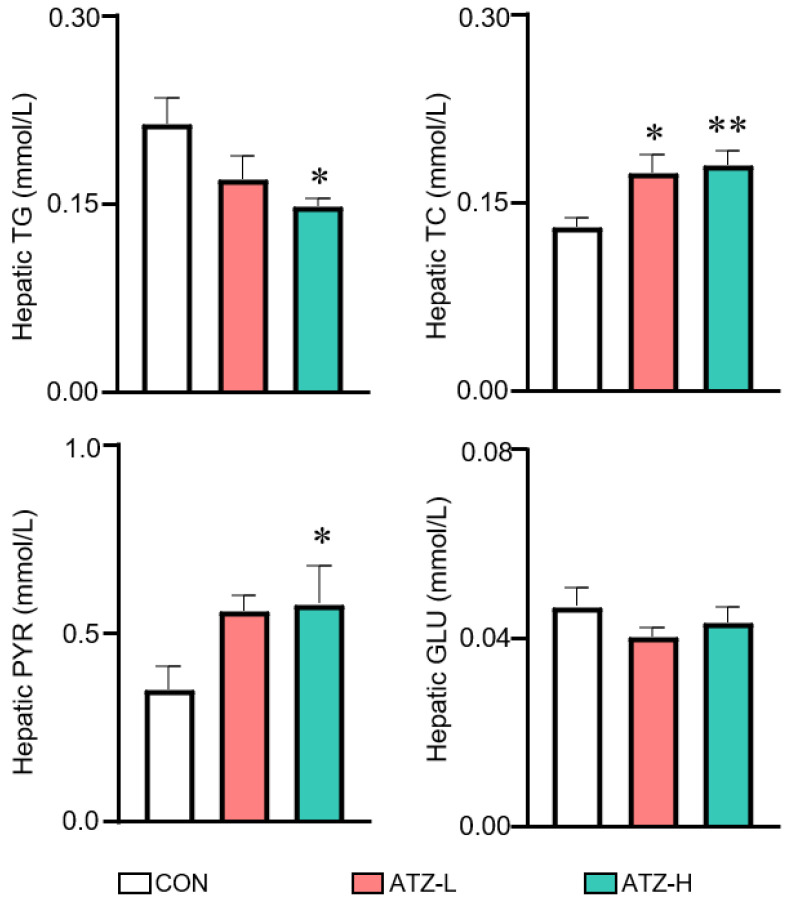
Changes in the levels of TG, TC, PYR and GLU in the liver of zebrafish treated with different concentrations of ATZ for 21 days. ATZ−L and ATZ−H represent 300 and 1000 μg/L ATZ, respectively. The presented values are the means ± SEMs (*n* = 6). Significant differences between the control and ATZ−treated groups were indicated with an asterisk (* *p* < 0.05, ** *p* < 0.01).

As shown in Figure 1, the levels of TG, TC and PYR were significantly (*p* < 0.05) altered in ATZ−exposed zebrafish compared to the control group. Compared with the control group, TG level in the ATZ-H group decreased significantly (*p* < 0.05), while the PYR level increased significantly (*p* < 0.05). The TC level notably increased after ATZ treatment. All these results indicated that ATZ could cause disorders of hepatic energy metabolism in zebrafish.

### 3.2. Metabolomic Alterations Induced by ATZ in the Liver

**Figure 2:**
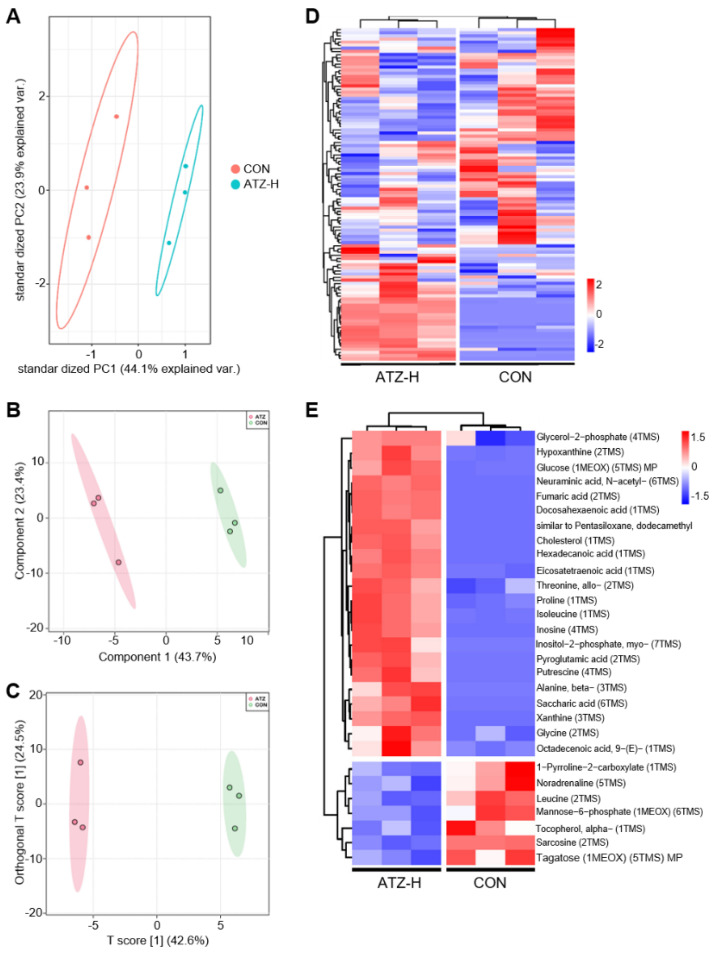
ATZ exposure altered hepatic metabolism in male adult zebrafish. (**A**) Score plots of principal component analysis (PCA) for 1000 μg/L of ATZ and the control group. (**B**) The PLS−DA and OPLS−DA. (**C**) Analyses of the control group and ATZ−treated group. (**D**) Heat map of liver metabolites in the control and 1000 μg/L ATZ−treated groups. (**E**) Heat map of significantly (*p* < 0.05) different metabolites in the control and ATZ−treated groups.

The hepatic metabolic profile in male zebrafish was also influenced in zebrafish after 21 days of exposure to ATZ (Figure 2). The PCA model was used to compare the metabolic differences in zebrafish liver between the control and ATZ-H group. In addition, the PCA score map showed that there was a significant separation of metabolites between the two groups (Figure 2A). Differences in metabolites between the two groups were screened using PLS−DA and OPLS−DA multidimensional analysis (VIP > 1, *p* value < 0.05). A significant deviation of metabolites occurred in the ATZ−H group compared to the control group (Figure 2B,C). The heat map in Figure 2D showed the overall metabolism of the two groups of metabolites. In addition to 29 metabolites that were significantly (*p* < 0.05) different between the control group and the treated group, there were many substances that were not identified. All these changed substances were enriched in many different metabolic pathways (Figure 2D). Different metabolic pathways were interrelated. A substance may participate in multiple metabolic pathways; thus, we believed that even if some substances would not change, one or more metabolic pathways they participate in may change. Moreover, we further screened out the differential metabolites between the two groups and plotted the heat map (Figure 2E). There were 29 significantly (*p* < 0.05) different metabolites in total. The results showed that for the group treated with 1000 μg/L ATZ, the levels of Glycerol−2−phosphate, Hypoxanthine, Glucose (1MEOX) (5TMS) MP, Neuraminic acid, N−acetyl− (6TMS), Fumaric acid (2TMS), Docosahexaenoic acid (1TMS), similar to Pentasiloxane, dodecamethyl, Cholesterol (1TMS), Hexadecanoic acid (1TMS), Hexadecanoic acid (1TMS), Threonine, allo− (2TMS), Proline (1TMS), Isoleucine (1TMS), Inosine (4TMS), Inositol−2−phosphate, myo− (7TMS), Inositol−2−phosphate, myo− (7TMS), Putrescine (4TMS), Putrescine (4TMS), Saccharic acid (6TMS), Xanthine (3TMS), Glycine (2TMS) and Octadecenoic acid, 9− (E)− (1TMS) were significantly higher than those of the control group, while 1-Pyrroline−2−carboxylate (1TMS), Noradrenaline (5TMS), Leucine (2TMS), Mannose−6−phosphate (1MEOX) (6TMS), Tocopherol, alpha− (1TMS), Sarcosine (2TMS) and Tagatose (1MEOX) (5TMS) MP were obviously lower compared to the control group. These results suggested that ATZ significantly (*p* < 0.05) altered the hepatic metabolism in zebrafish.

### 3.3. The Main Metabolic Pathways That Were Significantly Altered in ATZ−Exposed Zebrafish

**Figure 3:**
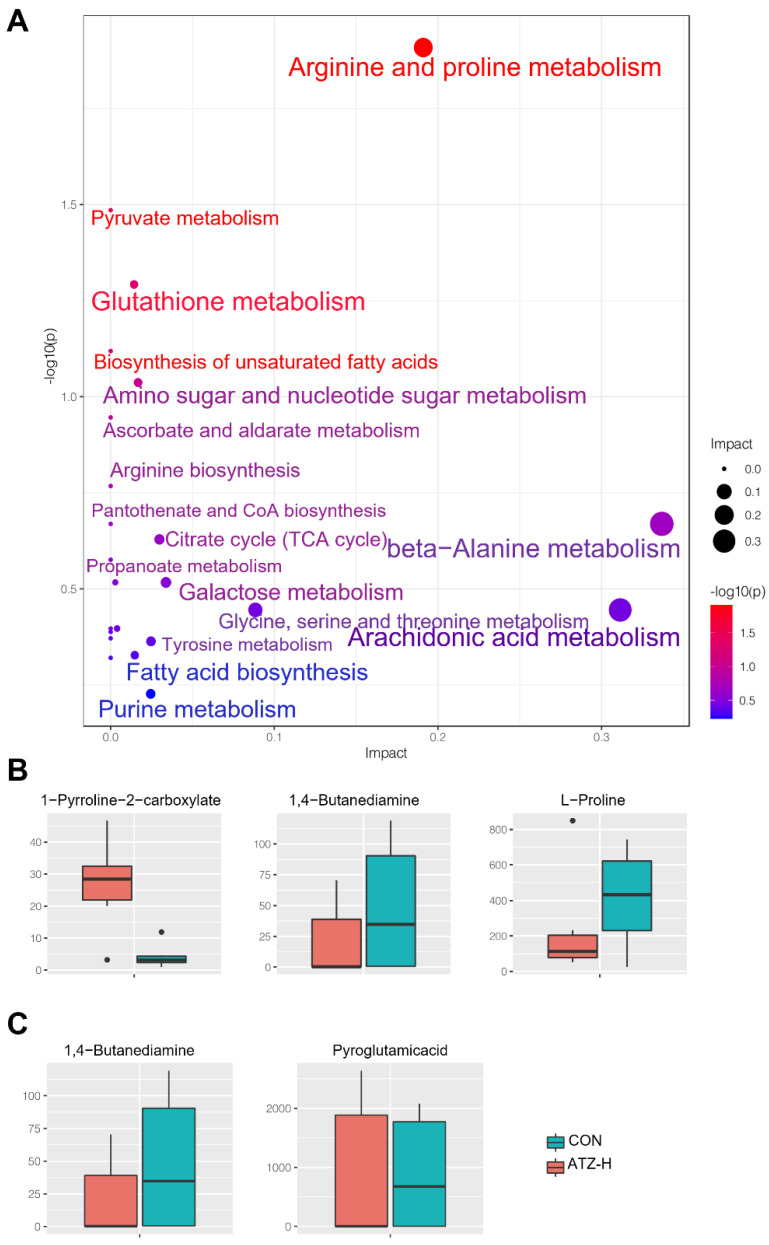
ATZ exposure induced enrichment and changes in different pathways of altered metabolites in zebrafish liver. (**A**) Pathway enrichment of some altered metabolites in the liver of zebrafish after 21 days of ATZ exposure. (**B**) Metabolites with major changes in the metabolic pathway of arginine and proline. (**C**) The main metabolites that change in the metabolic pathway of cysteine.

ATZ exposure induced alterations in hepatic metabolic pathways in zebrafish. The greatest effects were observed on the arginine and proline metabolic pathways (Figure 3A). In addition, the metabolic pathway of glutathione had been notably altered. Further analysis of these two metabolic pathways revealed that three main substances were significantly altered in the arginine and proline metabolic pathways, and they were 1-Pyrroline−2−carboxylate, 1,4−Butanediamine and L-Proline (Figure 3B). Compared with the control group, 1−Pyrroline−2−carboxylate in the ATZ−H group increased significantly (*p* < 0.05), while 1,4-Butanediamine and L-Proline decreased notably. Meanwhile, after 1000 μg/L ATZ exposure, the 1,4-Butanediamine and Pyroglutamicacid levels of the glutathione metabolic pathway were elevated (Figure 3C).

### 3.4. ATZ Induced the Changed Metabolites Involved in Different Pathways

**Figure 4:**
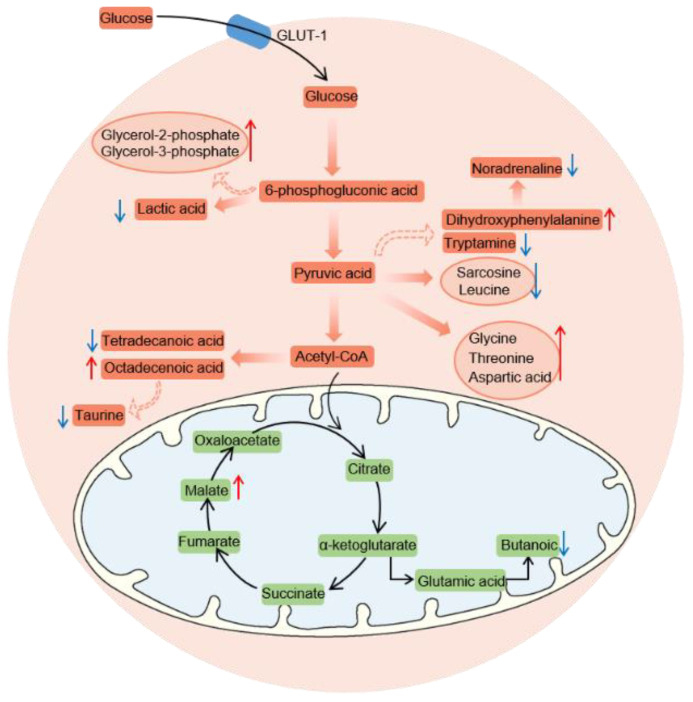
ATZ exposure leads to changes in glucose, lipid and amino acid related metabolites. Metabolites that changed significantly in the ATZ treatment group were indicated by different colored arrows. Red arrows indicate increased metabolite levels and blue arrows indicate decreased metabolite levels.

By analyzing the changes in metabolites, we found that ATZ caused changes in glycolipid metabolism, amino acid metabolism and some other related metabolic pathways (Figure 4). We found significant decreases in Tagatose and Mannose−6−phosphate, which directly affected the synthesis and breakdown of 6-phosphogluconic acid, Pyruvic acid and Acetyl−CoA. Malate involved in the TCA cycle was also significantly elevated. Other metabolites, whose levels were increased by ATZ, were Glycerol−2−phosphate, Glycerol−3−phosphate, Octadecenoic acid, Dihydroxyphenylalanine and Threonine. In contrast, the substances, whose levels decreased after exposure to ATZ, were Lactic acid, Tetradecanoic acid, Taurine, Noradrenaline, Tryptamine, Sarcosine, Leucine and Butanoic. Collectively, it could be found that ATZ might interfere with substances of different metabolic pathways.

## 4. Discussion

Increasingly more studies have suggested that environmental chemicals including pesticides had the potential to influence the hepatic metabolism in animals [35,36,37]. The extensive use of ATZ has made it easy for people and wildlife to come into contact with it. Due to its widespread use, ATZ has been detected in areas including agricultural land, where it can penetrate surface water [19]. Different studies have shown that the toxicity of ATZ was focused on endpoints such as endocrine disruption and oxidative stress [38,39]. ATZ has also been reported to be hepatotoxic in some experiments [40,41]; however, whether the ATZ could disturb the hepatic metabolism in zebrafish has not received attention. This study found that ATZ exposure for 21 days resulted in changes in a number of metabolites in the liver of zebrafish.

Metabolomics is becoming a popular approach in the study of organismal metabolism [42]. It can represent the dynamic changes of the system caused by physiological or pathological stimuli well. The traditional approach to toxicological studies only relates the toxicity results to the corresponding chemicals [43]. Here, we therefore analyzed whether ATZ affects liver metabolism by GC/MS−based metabolomics. The liver is an important site where the metabolism of exogenous substances in the body occurs. Cytochrome P450 and other enzymes are essential and important players in the process of metabolism in the liver [44]. In addition, the liver is an important site for many physiological processes to take place properly. These processes include nutrient metabolism, blood regulation and the regulation of cholesterol and lipid homeostasis [45]. The processing and metabolic processes of nutrients provide the body with energy, which is one of the liver’s most important functions. The liver could oxidize lipids and excess lipids can be stored as fat [46]. Meanwhile, proteins and amino acids cannot be metabolized without the liver’s metabolic processing [47]. 

The hypothesis that environmental chemical pollutants may cause metabolic abnormalities in the liver has been confirmed by many studies [37,41,48]. Previous studies have reported that exposure to streptozotocin can cause the normal metabolism of branched-chain amino acids in juvenile zebrafish [49]. Triglyceride is an important constituent of adipose tissue. In Figure 1, the significant decrease in the liver triglyceride level indicates that ATZ may affect lipid metabolism [50]. Meanwhile, total cholesterol is the main component of cell membrane, and it is also an important raw material for the synthesis of physiologically active substances such as the adrenocortical hormone, sex hormone, vitamin D and bile acid. High levels of total cholesterol in the liver can cause atherosclerosis and cardiovascular disease [51]. Pyruvate is a product of glycolysis and is involved in metabolic pathways such as catabolism, energy metabolism and anabolism. Under aerobic conditions, pyruvate enters the TCA cycle to participate in energy metabolism, and under anaerobic conditions, it accumulates and thus produces acid fermentation products. Here, high levels of pyruvate in the treatment group may cause acidosis [52]. Changes in the levels of these different substances indicate impaired hepatic glycolipid metabolism. Amino acids are important precursors for peptide and protein synthesis. In addition, many amino acids themselves play an important role. For example, amino acids play a specific function in the process of energy metabolism and neurotransmission. In recent years, the metabolic changes of amino acids have become an important means to detect diseases [53]. Gut microbes promote host resistance to hypothermia by stimulating arginine and proline metabolic pathways [54]. The important roles of glutathione are in nutrient metabolism, immune response, signal transduction and antioxidant [36]. Our results suggest that the liver is also a target organ of ATZ and may directly contribute to the disruption of liver metabolism in zebrafish. Interestingly, ATZ altered the normal levels of taurine, leucine and aspartic acid (Figure 4). Taurine is involved in the energy metabolism of several tissues and organs, including muscle, adipose tissue and the liver [55]. Leucine is essential for skeletal muscle development [56]. Aspartic acid has been implicated as a pathophysiological factor in certain diseases, such as atherosclerosis [57].

In this study, male adult zebrafish were exposed to 300 and 1000 g/L of ATZ for 21 days, respectively. We observed that 21 days of ATZ exposure could cause abnormal liver metabolism in zebrafish. These results showed that several different pathways related to metabolism could be influenced by ATZ, and the potential effects of ATZ on fish or other aquatic animals should be extensively studied. We thought that the results made in this study could provide new insights into the toxicity of ATZ in the aqueous environment.

## Data Availability

Not applicable.
